# Algal-Derived Synthesis of Silver Nanoparticles Using the Unicellular *ulvophyte* sp. MBIC10591: Optimisation, Characterisation, and Biological Activities

**DOI:** 10.3390/molecules28010279

**Published:** 2022-12-29

**Authors:** Reham Samir Hamida, Mohamed Abdelaal Ali, Mariam Abdulaziz Alkhateeb, Haifa Essa Alfassam, Maha Abdullah Momenah, Mashael Mohammed Bin-Meferij

**Affiliations:** 1 Nanobiology Lab, Institute for Protein Research, Osaka University, Osaka 565-0871, Japan; 2 Plant Production Department, Arid Lands Cultivation Research Institute, City of Scientific Research and Technological Applications (SRTA-CITY) New Borg El-Arab, Alexandria 21934, Egypt; 3 Department of Biology, College of Science, Princess Nourah bint Abdulrahman University, Riyadh 11671, Saudi Arabia; 4 Histopathology Unit, Research Department, Health Sciences Research Center (HSRC), Princess Nourah bint Abdulrahman University, Riyadh 11671, Saudi Arabia

**Keywords:** green synthesis, microalgae, anticancer, antibacterial, optimisation parameter

## Abstract

Algal-mediated synthesis of nanoparticles (NPs) is an eco-friendly alternative for producing NPs with potent physicochemical and biological properties. Microalgae represent an ideal bio-nanofactory because they contain several biomolecules acting as passivation and stabilising agents during the biogenesis of NPs. Herein, a novel microalgae sp. was isolated, purified, and identified using light and electron microscopy and 18s rRNA sequencing. The chemical components of their watery extract were assessed using GC-MS. Their dried biomass was used to synthesise silver (Ag) NPs with different optimisation parameters. Ag-NPs were physiochemically characterised, and their anticancer and antibacterial effects were examined. The data showed that the isolated strain was 99% similar to the unicellular *ulvophyte* sp. MBIC10591; it was ellipsoidal to spherical and had a large cup-shaped spongiomorph chloroplast. The optimum parameters for synthesising Ag-NPs by unicellular *ulvophyte* sp. MBIC10591 (Uv@Ag-NPs) were as follows: mixture of 1 mM of AgNO_3_ with an equal volume of algal extract, 100 °C for 1 h, and pH of 7 under illumination for 24 h. TEM, HRTEM, and SEM revealed that Uv@Ag-NPs are cubic to spherical, with an average nanosize of 12.1 ± 1.2 nm. EDx and mapping analysis showed that the sample had 79% of Ag, while FTIR revealed the existence of several functional groups on the NP surface derivatives from the algal extract. The Uv@Ag-NPs had a hydrodynamic diameter of 178.1 nm and a potential charge of −26.7 mV and showed marked antiproliferative activity against PC3, MDA-MB-231, T47D, and MCF-7, with IC_50_ values of 27.4, 20.3, 23.8, and 40 µg/mL, respectively, and moderate toxicity against HFs (IC_50_ of 13.3 µg/mL). Uv@Ag-NPs also showed marked biocidal activity against Gram-negative bacteria. *Escherichia coli* was the most sensitive bacteria to the NPs with an inhibition zone of 18.9 ± 0.03 mm. The current study reports, for the first time, the morphological appearance of the novel *unicellular ulvophyte* sp., MBIC10591, and its chemical composition and potential to synthesise Uv@Ag-NPs with smaller sizes and high stability to act as anti-tumour and microbial agents.

## 1. Introduction

Green synthesis has become a reliable and sustainable method for the biogenesis of several nanomaterials such as metallic nanoparticles (NPs) (M-NPs), metal oxide NPs, bimetallic NPs, and quantum dots [1]. NPs represent potent alternative drugs for several diseases, such as infectious diseases [2], cancers [3], diabetes [4], and wound healing [5], due to their unique features including their smaller size to larger surface area, various shapes, and sufficient reactivity facilitating their use for drug delivery, sensing, and catalysis, among others [6,7,8]. Generally, NPs are synthesised by three main methods: physical, chemical, and biological (green) syntheses [9]. Phycosynthesis is a green synthesis (bottom-up) route that uses algal cells and their biocomponents to produce NPs with various shapes and sizes [10]. Microalgae are considered model microorganisms for the biogenesis of NPs due to their potential to hyper-accumulate heavy metals and be redesigned to more malleable shapes [11]. Moreover, microalgae contain diverse biomolecules such as lipids, proteins, carbohydrates, vitamins, pigments (such as phycocyanin, chlorophyll, and carotenoids), antioxidants, and others that precipitate during the biogenesis of NPs as reducing and stabilising agents [12]. The *Chlorophyta* phylum includes several species that are sources of several secondary metabolites that act as new drugs in the nutraceutical and pharmaceutical industries [13]. The unicellular *ulvophyte* sp. MBIC10591 is a strain belonging to the *Chlorophyta* phylum. Unfortunately, there are no publications on its morphology and applications. This strain was isolated and deposited by Japanese scientists Suda et al. in GenBank for the first time in 2001 with the accession number AB058370. Several studies used microalgae and cyanobacteria to produce several types of metallic and metallic oxide NPs such as Au- [14], Ag- [15], ZnO- [16], and TiO_2_-NPs [17]. Hamida et al. synthesised hexagonal Ag-NPs using the novel microalgae strain *Coelastrella aeroterrestrica* BA_Chlo4 with a smaller diameter of 14.5 ± 0.5 nm [12]. The NPs showed marked activity against different tumour cells, including MCF-7 and MDA, HCT-116, and HepG2 cells, with low toxicity against the normal cells (HFs and Vero). They also demonstrated moderate antioxidant activity and marked biocidal activity against both Gram-positive and -negative bacteria. The biological synthesis method requires the adjustment of physicochemical and biological parameters to obtain M-NPs with controlled sizes, shapes, and dispersity. Several studies have reported that the precursor concentrations, reactant ratios, temperature, pH, reaction time, time of exposure, and illumination conditions are important factors that influence the physicochemical and biological properties [18,19,20]. It was found that the increase in precursor concentration caused an increase in NP intensity, suggesting polydispersity and agglomeration of NPs at higher concentrations [21]. The change in the temperatures of the NP synthesis process may result in the formation of smaller or larger NPs. It was found that the reduction in NP size at higher temperatures could be attributed to an increase in the nucleation kinetics constant instead of the decreased growth kinetics constant, considering the concentrations of the precursors [22]. Among M-NPs, Ag-NPs have more effectiveness against microbes and cancerous cells. Aziz et al. used *Chlorella pyrenoidosa* as a source of reducing and stabilising agents to fabricate Ag-NPs and found that the resultant biogenic NPs exhibited a marked antibacterial activity against *Klebsiella pneumoniae*, *Aeromonas hydrophila*, *Acenetobacter* sp., and *Staphylococcus aureus* [23]. Ag-NPs are also used in sunscreen lotions, burn treatments, wound dressings, textiles, dental materials, and bone implants [24,25,26]. The Ag-NP mechanisms inside living cells have been reported to depend on their potential to facilitate oxidative stress by promoting the formation of reactive oxygen species. Moreover, their small sizes, potential charge, and surface chemistry enable their interactions with cellular proteins and DNA, resulting in cellular growth inhibition and death [3,27,28]. The current study revealed, for the first time, the morphology and chemical components of the novel unicellular *ulvophyte* sp. MBIC10591 and its potential for the biogenesis of Ag-NPs under optimum conditions and anticancer and antibacterial activities.

## 2. Results and Discussions

### 2.1. Algal Identification

#### 2.1.1. Morphological Appearance

**Figure 1:**
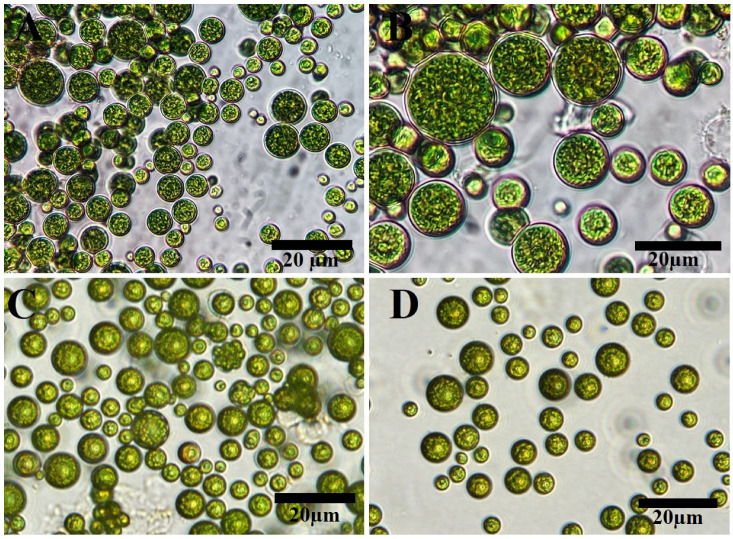
Light (**A**,**B**) and inverted light (**C**,**D**) microscopy of unicellular *ulvophyte* sp. MBIC10591. Scale bar = 20 µm.

**Figure 2:**
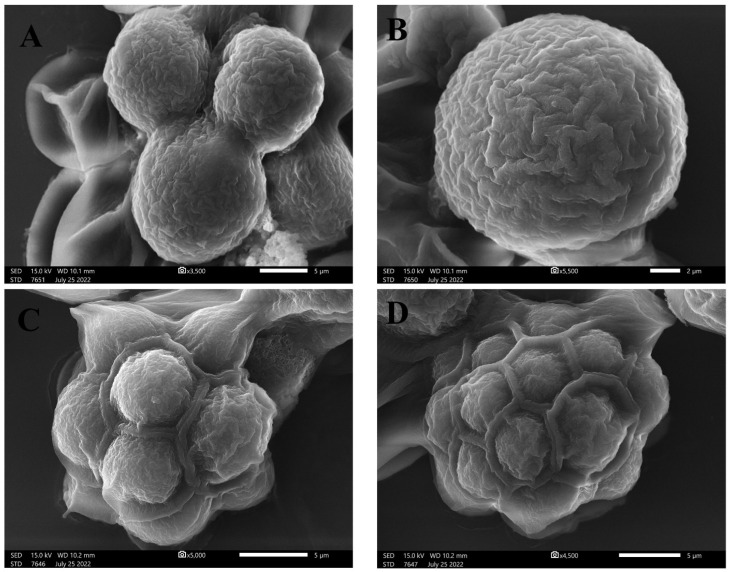
SEM micrographs of unicellular *ulvophyte* sp. MBIC10591 showing the morphology of single cells of *ulvophyte* sp. MBIC10591 (**A**,**B**) and package cells with a parenchyma-like structure containing more daughter cells (**C**,**D**). Scale bar = 5 µm (**A**,**C**,**D**) and 2 µm (**B**).

Light and inverted light micrographs revealed that the unicellular *ulvophyte* sp. MBIC10591 was spherical. Several unicellular vegetative cells were detected with cup-shaped spongiomorph chloroplasts with pyrenoids surrounded by several starch grains. Single cells were the most dominant; however, package cells with parenchyma-like structures containing more daughter cells were detected. All cells were surrounded by thick cell walls (Figure 1A–D). SEM micrographs showed cells with widely ellipsoidal to spherical shapes with sizes of 13.8 × 12.7 µm. Several irregular ribs existed on algal surfaces, and parenchyma-like structure package cells with more than nine daughter cells were observed (Figure 2A–D). The daughter cells were surrounded by thick cell walls. Unfortunately, no publications have demonstrated the morphological appearance of this isolate. The strain was deposited in 2001 for the first time by Japanese scientists Suda et al. in GenBank with the accession number AB058370. However, the current isolate shared several features with *Desmochloris* sp. in that its cells are distinguished by their spherical to ellipsoidal shapes and cup-shaped spongiomorph chloroplasts [29,30].

#### 2.1.2. Molecular Identification

**Figure 3:**
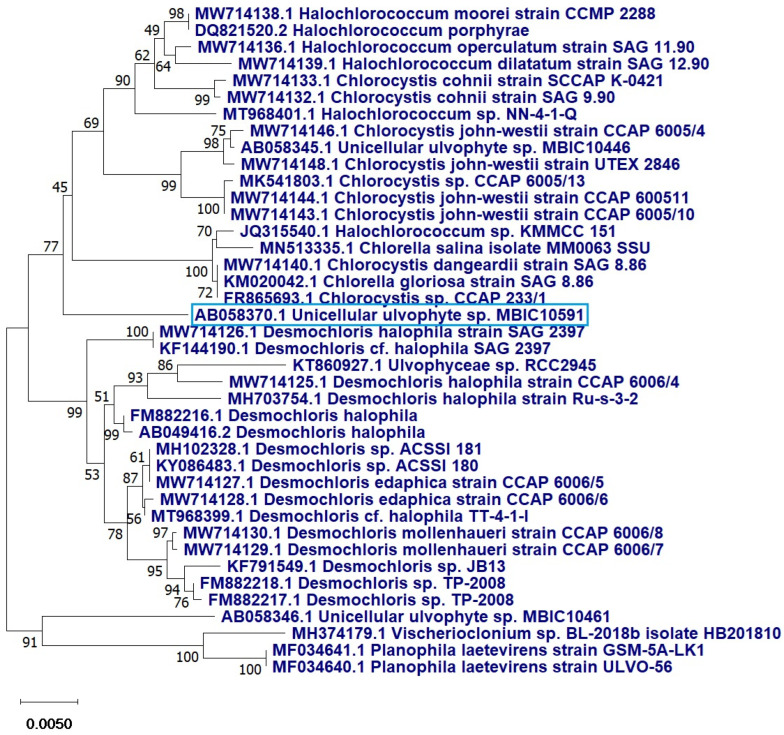
Phylogenetic tree of the unicellular *ulvophyte* sp. MBIC10591 (blue frame) inferred from 18S r RNA. Tree was constructed by cluster method using MEGA4 software version 10.2.6. Number at each branch refers to the bootstrap values for % of 1000 replicate trees calculated by neighbour joining statistical method.

The 18s rRNA analysis revealed that the current strains were 99% similar to the unclassified unicellular *ulvophyte* sp. MBIC10591 with a query covering of 89%. The sequence was deposited in GenBank, NCBI, with accession number OP605382. The phylogenetic tree demonstrated that the unicellular *ulvophyte* sp. MBIC10591 may be clustered within *Desmochloris* sp. (Figure 3).

#### 2.1.3. GC-MS Analysis

**Figure 4:**
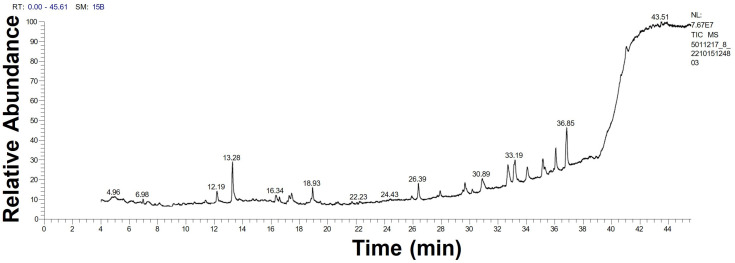
GC-MS chromatogram of unicellular *ulvophyte* sp. MBIC10591 watery extract.

The GC-MS chromatograph demonstrated, for the first time, the volatile organic molecules of the watery unicellular *ulvophyte* sp. MBIC10591 extract with a retention time of 4–44 min. The data showed 34 peaks corresponding to 24 algal bio-compounds. These 24 biomolecules included fatty acids (FA), FA esters, vitamins, alcohols, phenols, hydrocarbons, organosulphur compounds, amino-acid-like compounds, and polysaccharides (Table 1 and Figure 4). It was found that the unicellular *ulvophyte* sp. MBIC10591 was enriched with various molecules that act as antioxidant, antimicrobial, anticancer, and anti-hypercholesterolemic agents such as D-fructose, diethyl mercaptal, pentaacetate, 25,26,27-trinorcholecalcifer-24-al, trisulphide, and di-2-propenyl, among others [30,31,32]. Based on the GC-MS spectra, the main organic molecules were speculated to be lipids and hydrocarbons that could precipitate while stabilising NPs. However, the existence of alcohols and phenols may indicate that hydroxyl groups have significant roles in the biogenesis of Uv@Ag-NPs. Olasehinde et al. analysed the ethanolic and dichloromethane extracts of *Chlorella sorokiniana* and *Chlorella minutissima* and found that the microalgae were enriched with phenols, sterols, steroids, fatty acids, and terpenes that have modulatory activities for some mediators of Alzheimer’s disease [33]. GC-MS analysis of the aqueous extract of *Coelastrella aeroterrestrica* BA_Chlo4 showed that the dominant biomolecules of the algal extract were fatty acids and hydrocarbons [12].

### 2.2. Uv@Ag-NPs Synthesis

#### 2.2.1. Optimisation Parameters of Uv@Ag-NPs Synthesis

**Figure 5:**
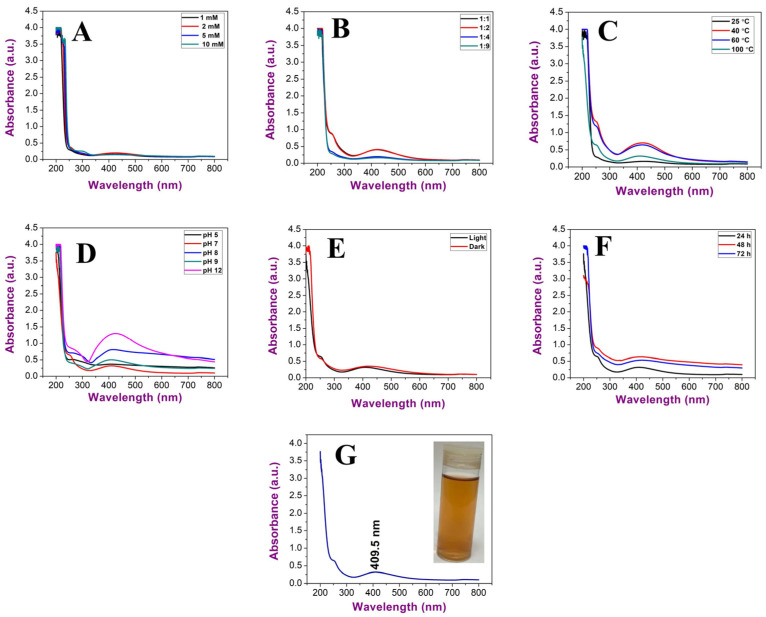
UV–Vis spectroscopy graphs illustrating the influence of (**A**) AgNO_3_ concentration, (**B**) ratio between algal extract and AgNO_3_, (**C**) temperature, (**D**) pH, (**E**) illumination conditions, and (**F**) incubation duration and (**G**) Uv@Ag-NPs under optimum conditions.

To obtain smaller nanoparticles with high stability, various parameters were studied, including precursor concentrations, the ratio between algal extract and precursor, temperature, pH, illumination, and time of incubation (Figure 5A–G).

The data revealed an increment in wavelengths of Uv@Ag-NPs from 1 mM (425 nm) and 2 mM (425.5 nm) to 5 mM (428 nm) at a constant ratio of 1:9 of algal extract to AgNO_3_, temperature of 25 °C, pH of 7, and light illumination for 24 h. On the other hand, with 10 mM of AgNO_3_, no NPs were produced, suggesting that the higher concentrations above 5 mM significantly slowed the generation of nuclei and growth down. Therefore, it took a longer time to complete the reduction in precursors. The concentration of the NP in their suspension at 1, 2, and 5 mM was low, and the suspension had a faint golden-yellow colour. The data revealed that 1 mM of AgNO_3_ was the optimum for Uv@Ag-NP synthesis. Khan et al. showed that the intensity of Ag-NPs synthesised from the *Piper betle* leaf extract increased at higher concentrations of 3 and 4 mM with high wavelength values relative to the other lower concentrations of 1 and 2 mM of AgNO_3_; this suggests polydispersity and agglomeration of Ag-NPs at higher concentrations [21]. Changing the ratio of the algal extract to AgNO_3_ from 1:9 to 1:1, 1:2, and 1:4 at a constant 1 mM AgNO_3_ caused a reduction in wavelength from 425 nm at a 1:9 ratio to 422, 422, and 422.5 nm, respectively, suggesting that a higher volume of the precursor may result in an increase in the NP size or promote the agglomeration of NPs [34]. An increase in the temperature during the biofabrication of Uv@Ag-NPs resulted in reductions in the wavelength from 422 nm at 25 °C to 420 nm at 40 °C 418.5 nm at 80 °C and 409.5 nm at 100 °C. The NP intensity was higher at both 40 and 80 °C; however, their peaks were broader, which indicated the synthesis of larger or agglomerated NPs. These data suggested that the higher temperature was an important parameter for the biogenesis of Uv@Ag-NPs. This could be attributed to the existence of algal biomolecules that become activated at higher temperatures during Uv@Ag-NPs synthesis or kinetic influence. Liu et al. reported that the reduction in NP sizes at higher temperatures could be attributed to an increase in the nucleation kinetics constant instead of the decreased growth kinetics constant, considering the concentrations of the precursors [22]. UV–Vis spectroscopy showed that acidic pH (5) resulted in a broader SPR peak with a wavelength of 408.5 nm. However, the colour of the NP suspension was transparent, suggesting a slower synthesis reaction with a low yield of UV@Ag-NPs. Moreover, the wavelength of the Uv@Ag-NPs at pH of 7 (the same as the original pH of the reaction without any adjustment) and 9 was 409.5 nm; at higher pH values (8 and 12), the wavelengths shifted from 409.5 nm to 418 and 428 nm, respectively. These data explained that the pH values of 7 and 9 were suitable for producing smaller Uv@Ag-NPs, while increasing the pH to 8 and 12 caused an increase in NP intensity with wide shifting in wavelength indicating the synthesis of larger NPs. These data could be explained by the influence of pH on the dissociation, isolation, interfacial free energy, and the net charge of NPs. For instance, in an acidic medium, the driving force of NP dissolution may be balanced by the repulsive force keeping the dispersion of NPs resulting in smaller NPs. On the contrary, the negative charge hydroxyl ions (OH^-^) facilitated the reduction of silver ions to NPs by increasing the ion levels in the medium silver atoms; these tend to diffuse between adjacent adsorption sites on a surface and form bonds with nearest neighbour atoms via Brownian diffusion, resulting in the formation of larger NPs [35]. Traiwatcharanon et al. synthesised Ag-NPs using a *Pistia stratiotes* extract and studied the influence of pH on NP size [35]. They reported that acidic conditions at pH values of 4, 5, and 6 caused blue shifting in the SPR of the Ag-NPs with smaller wavelengths of 330 nm while resulting in red shifting of SPR peaks with a wavelength of 414 nm. They reported that the red shift in the basic medium suggests larger Ag-NPs with higher intensities than those generated under acidic and neutral conditions.

The data showed that the optimum illumination condition for Uv@Ag-NP synthesis was under light (409.5 nm); under dark conditions, their wavelength was 420 nm. Increasing the duration of incubation from 24 h to 72 h under illumination increased the wavelength values from 409.5 to 421.5 nm, respectively, suggesting that the duration of exposure to light influences NPs stability. This could be attributed to the photocatalytic reaction where photons produce energetic electrons that excite SPR and, as a result, reduce Ag^+^ to Ag-NPs [35,36,37]. However, high exposure to light irritation may accelerate the agglomeration rate of NPs. Husain et al. synthesised silver nanoparticles using 30 cyanobacteria species under dark and light conditions and found that almost all species were able to generate Ag-NPs only under light conditions [38].

Based on the previous data, the optimum conditions for synthesising Uv@Ag-NPs were 1 mM AgNO_3_, 1:1 ratio of AgNO_3_ and algal extract, temperature of 100 °C for 1h, pH of 7, light conditions, and incubation duration of 24 h. These conditions resulted in golden brown NP suspension at a wavelength of 409.5 nm. Kusumaningruma et al. reported that the maximum SPR peak of biosynthesised Ag-NPs using *Chlorella pyrenoidosa* was at 410 nm, which confirms the nanostructure of Ag-NPs [39].

#### 2.2.2. Uv@Ag-NPs Characterisations

##### TEM, SEM, EDx, and Mapping Analysis

**Figure 6:**
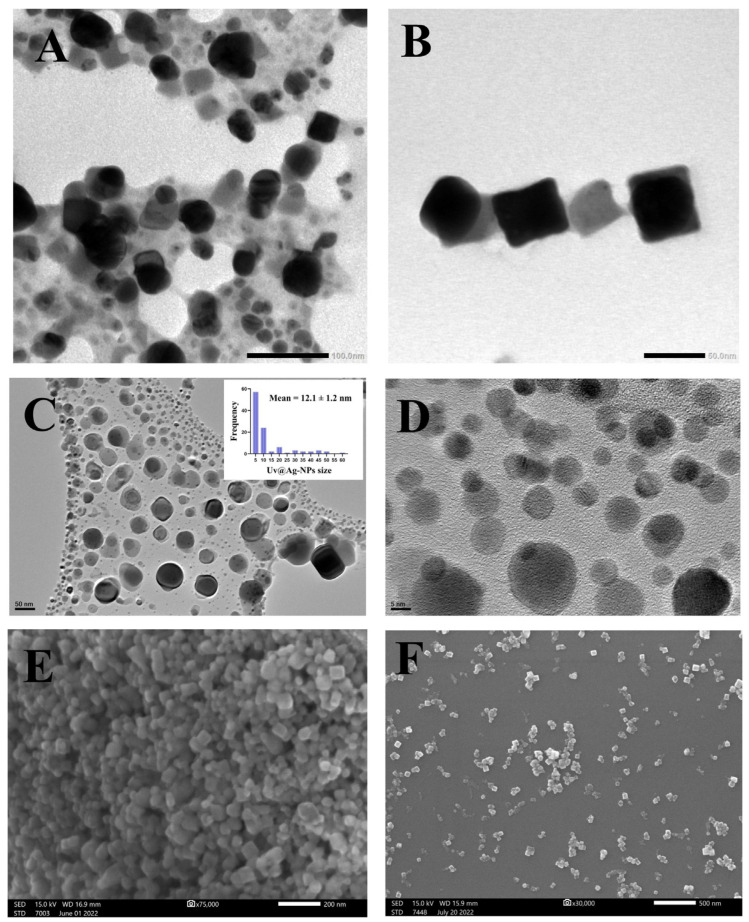
TEM (**A**,**B**), HR-TEM (**C**,**D**), and SEM (**E**,**F**) micrographs of Uv@Ag-NPs illustrate the uniform distribution of Uv@Ag-NPs and their spherical and cubic shapes. Scale bar = 100 nm (**A**), 50 nm (**B**,**C**), 5 nm (**D**), 200 nm (**E**), and 500 nm (**F**).

The TEM, HR-TEM, and SEM micrographs (Figure 6) of the Uv@Ag-NPs showed that the NPs had polyform shapes, including spherical and cubic. These NPs were trapped in an algal matrix that could contain polysaccharides. Smaller spherical Ag-NPs and cubic NPs may represent the seed for generating cubic NPs [40].

The micrographs also demonstrated that Uv@Ag-NPs were uniformly distributed without agglomeration, suggesting that Uv@Ag-NPs have good stability. The frequency distribution analysis of Uv@Ag-NPs using HR-TEM micrographs suggested that Uv@Ag-NPs are small, with a nanosize range of 5–60 nm and an average diameter of 12.1 ± 1.2 nm. Kannan et al. fabricated silver nitrate using the *Chlorophyceae Codium capitatum P.C. Silva* strain and showed that Ag-NPs have a cubic shape with a nanosize range of 3–44 nm and a mean diameter of 30 nm [41].

**Figure 7:**
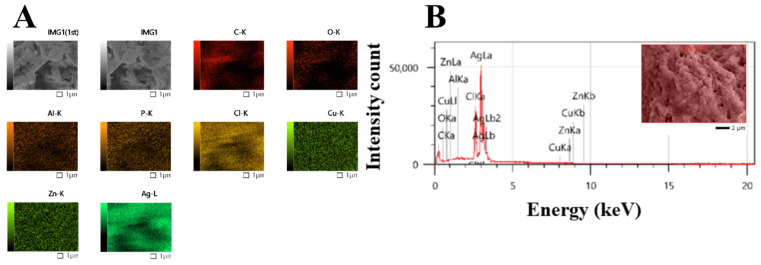
Map (**A**) and EDx (**B**) analysis of Uv@Ag-NPs synthesised from the unicellular *ulvophyte* sp. MBIC10591.

The elemental compositions of Uv@Ag-NPs and their distribution were determined using the EDx detector. The data showed that the main element distributed in the sample was Ag. A sharper peak was detected at 3 keV, which is a typical absorption signal of Uv@Ag-NPs with a mass percentage of 76.7%. Other elements, including carbon (6.93%), oxygen (1.81%), and chloride (12.18%), were detected while other trace elements emerged, including aluminium (0.3%), copper (1.13%), and zinc (0.91%); they may have emerged from the algal biocompounds surrounding the NPs or they existed in the polysaccharide matrix (Table 2, and Figure 7A,B) [39,42].

##### FTIR

**Figure 8:**
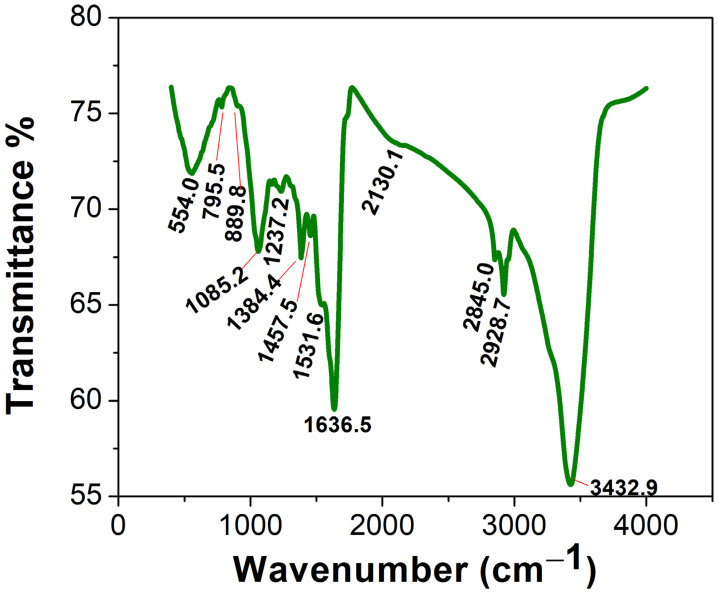
FTIR spectra of the Uv@Ag-NPs synthesised using the unicellular *ulvophyte* sp. MBIC10591.

The FTIR of the Uv@Ag-NPs contained 13 peaks at 3432.9 [41], 2928.7 [43], 2845.0 [44], 2130.1, 1636.5 [45], 1531.6, 1457.5 [46], 1384.4 [47], 1237.2 [48], 1085.2 [49], 889.8, 795.5 [50], and 554.0 [51] cm^−1^ (Figure 8). The IR peaks at 3432.9, 2928.7, and 2845.0 cm^−1^ corresponded to strong broad O-H stretching of alcohols or medium N-H stretching of primary amines and strong broad O-H stretching of carboxylics, broad N-H stretching of amine salts, or medium C-H stretching of alkane. However, the peaks at 2130.1, 1636.5, and 1531.6 cm^−1^ referred to the strong N=N=N stretching of azides, N=C=N stretching of carbodiimides, or N=C=S stretching of isothiocyanates or weak CΞC of alkynes; medium C=C stretching of alkenes or N-H stretching of amines; and strong N-O stretching of nitrocompounds. The peaks at 1457.5, 1384.4, 1237.2, and 1085.2 cm^−1^ were related to the medium C-H bending of alkanes; medium C-H bending of alkanes, O-H bending of alcohols, or strong S=O stretching of sulphates; strong C-O stretching of alkyls or medium C-N stretching of amines; and strong C-O stretching of primary alcohols or aliphatic ethers. The FTIR spectra at 889.8, 795.5, and 554.0 cm^−1^ were related to strong or medium C=C bending of alkenes and strong C-I stretching of halocompounds. These data may indicate that the main molecules for capping Uv@Ag-NPs were proteins and/or polysaccharides and/or alcohols, while the stabilising molecules were hydrocarbons and/or fatty acids. These data may be supported by the data of GC-MS analysis, which indicated that the main stabilising agents were fatty acids and hydrocarbons, and that phenol, alcohols, and/or amino-acid-like compounds were the reducing agents. Mahajan et al. extracellularly biofabricated Ag-NPs from silver nitrate using *Chlorella vulgaris* [52]. They analysed the functional group on the Ag-NPs using FTIR and found that the IR peaks of Ag-NPs were at 3435.88, 2092.30, 1637.82, 1559.61, 1414.42, 1037.17, and 618.16 cm^−1^. This suggested that proteins, polysaccharides, and amides were significant passivating biomolecules for the bioreduction of AgNO_3_ to Ag-NPs, while long-chain fatty acids were the stabilising agents.

##### DLS and Zeta Potential

**Figure 9:**
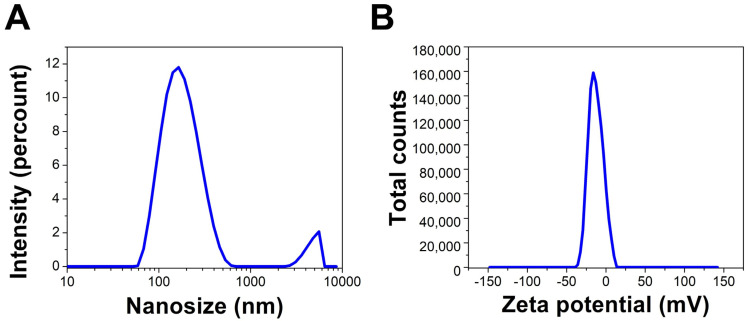
DLS (**A**) and zeta potential (**B**) of Uv@Ag-NPs synthesised from the unicellular *ulvophyte* sp. MBIC10591.

The hydrodynamic diameter (HD) average of the Uv@Ag-NPs in an aqueous system was 178.1 nm with a polydispersity index of 0.38, suggesting that Uv@Ag-NPs had a polydisperse standard. The larger NP sizes than the nanosize range 5–60 nm calculated using the HR-TEM micrographs could be attributed to the algal biomaterials in the suspension and surrounding the surface of NPs; they tend to absorb water molecules on the NP surfaces, which increases the HD. The zeta potential (ZP) of the NPs is important for understanding their degree of stability in colloidal systems. NPs with higher negativity or positivity have strong repletion forces to repel each other, which prevents the agglomeration of NPs and stabilises them in a colloidal system [53]. Ardani et al. reported that the ZP value range of ±0–10 mV indicates a highly unstable colloid, while the ranges of ±10–20 mV, ±20–30 mV, and >±30 mV reveal relatively, moderately, and highly stable colloids, respectively [53]. The ZP of the Uv@Ag-NPs was −26.7 mV, indicating colloidal stability. This negative charge surrounding Uv@Ag-NPs could be normalised to those of the algal functional groups, such as hydroxyl and carboxylic groups, which surround the surfaces of NPs. Rathod et al. reported that the ZP of Ag-NPs synthesised from the *Nocardiopsis valliformis strain OT1* was −17.1 mV, suggesting their colloidal stability (Figure 9A,B) [54].

### 2.3. Antiproliferative Effect of Uv@Ag-NPs

Uv@Ag-NPs significantly reduced the proliferative activity of PC3, MDA-MB-231, T47D and HFs cell lines in a dose-dependent manner. However, MCF-7 cells responded differently to Uv@Ag-NPs. Uv@Ag-NPs drastically inhibited cellular proliferation in a dose-dependent manner from 200 to 50 µg/mL. The cell viability was non-significantly increased at 25 to 6.25 µg/mL of Uv@Ag-NPs. Interestingly, 3.13 µg/mL of Uv@Ag-NPs significantly reduced MCF-7 cell growth by 22%, whereas 1.5 µg/mL of Uv@Ag-NPs demonstrated a non-significant reduction in the malignant cell activity by 12%. This may be explained by the way that drug-responsive malignant cells behave or by the fact that smaller NPs can enter cells at lower concentrations since there are fewer aggregates present.

**Figure 10:**
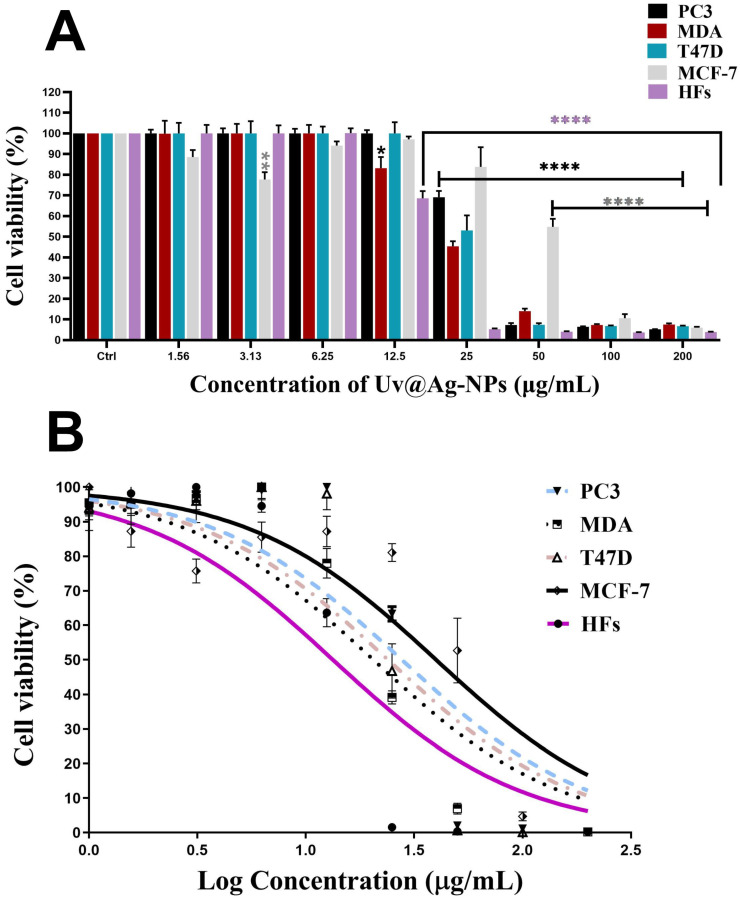
Antiproliferative activity (**A**,**B**) of a twofold serial dilution of 200 µg/mL of Uv@Ag-NPs synthesised from the unicellular *ulvophyte* sp. MBIC10591 against four malignant cells, PC3, MDA-MB-231, T47D, and MCF-7, and normal cells, HFs. Data are represented as mean ± SEM. *p*-values were calculated versus untreated cells: **** *p* < 0.0001, ** *p* < 0.001, and * *p* < 0.01.

**Figure 11:**
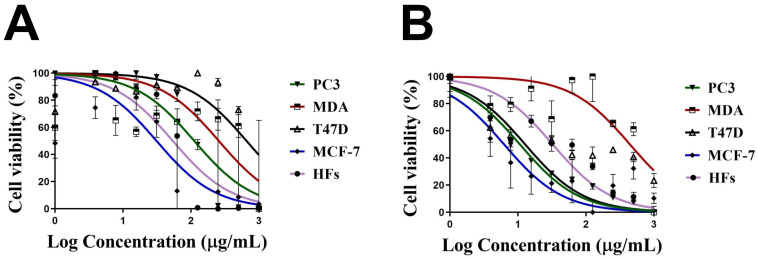
Cell viability of 1000 µg/mL of chemically synthesised Ag-NPs (Ch@Ag-NPs) (**A**) and 5-fluorouracil (5-FU) (**B**) against four malignant cells, PC3, MDA-MB-231, T47D, and MCF-7, and normal cells, HFs. Data are represented as mean ± SEM.

Similarly, the cell viability % of HFs was significantly decreased by increasing the Uv@Ag-NPs concentration from 12.5 to 200 µg/mL. Beyond 12.5 µg/mL, there was no significant activity of NPs against HFs cells. The moderate toxicity of Uv@Ag-NPs against HFs cell lines could be attributed to the algal functional groups surrounding the NPs, which have antioxidant activity, as reported in the GC-MS analysis section, increasing the NPs’ biocompatibility against normal cells. These data suggested that Uv@Ag-NPs may act as potent alternative drugs for traditional therapeutic agents or pharmaceutical applications. The IC_50_ values of Uv@Ag-NPs against PC3, MDA-MB-231, T47D, MCF-7, and HFs were 27.4, 20.3, 23.8, 40.0, and 13.3 µg/mL (Figure 10). These data revealed that the most sensitive malignant cells to Uv@Ag-NPs were MDA-MB-231, followed by T47D, PC3, and MCF-7 cells. This suggested that Uv@Ag-NPs could be used as antiproliferative agents against prostate and multidrug-resistant breast cancer cell lines. The great antiproliferative activity of Uv@Ag-NPs against MDA-MB-231 cells compared to other cells may be attributed to the cellular metabolic state influencing cellular charge and their interaction with the charged NPs. On the other hand, the IC_50_ values of Ch@Ag-NPs against PC3, MDA-MB-231, T47D, MCF-7, and HFs were 111.8, 256.9, 657.0, 31.2, and 54.1 µg/mL, while the IC_50_ values of 5-FU against PC3, MDA-MB-231, T47D, MCF-7, and HFs were 10.6, 442.27, 12.75, 56.48, and 32.4 µg/mL (Figure 11). These data indicated that Uv@Ag-NPs demonstrated marked activity against tested cancer cells relative to other tested drugs, including Ch@Ag-NPs and 5-FU. The marked activity of Uv@Ag-NPs against cancer cells may be attributed to their smaller size, which facilitates penetration of cell boundaries and interactions with biomolecules, including proteins, enzymes, and antioxidants, causing cellular dysfunction and cell death [55]. Moreover, the bio-functional group derivatives from the algal components may play a significant role in enhancing the antiproliferative effects of Uv@Ag-NPs; they may facilitate the transport of NPs within cells via interactions with cellular receptors. Moreover, the negative charge on Uv@Ag-NPs may influence the therapeutic activity of NPs by increasing the attractive force between NPs and the cellular membrane and the resultant increase in the adsorption of NPs on the cellular surface. This surges the probability of these NPs moving inside cells and interacting with cell membranes [56]. Mohanta et al. synthesised Ag-NPs using *Gracilaria edulis* and found that Ag-NPs (with an average diameter of 62.7 ± 0.25 nm and a spherical shape) caused 50% death of MDA-MB-231 cells at concentrations of 344.27 ± 2.56 µg/mL, suggesting the potent antiproliferative activity of NPs [57]. Ag-NPs (with nanosize of 5–50 nm and spherical shape) synthesised from *Pleurotus djamor var. roseus* exhibited antiproliferative activity against PC3 cells with an IC_50_ of 10 µg/ mL [58], while Ag-NPs (with spherical shape and size range of 5–25 nm) synthesised from *Anabaena flos-aquae* reduced 50% of T47D cell growth with an IC_50_ of 5 µg/ mL [59]. Hexagonal Ag-NPs synthesised from the novel *Coelastrella aeroterrestrica* strain BA_Chlo4 with a diameter of 14.5 ± 0.5 nm showed marked inhibitory activity against MCF-7, MDA-MB-231, HCT-116, HepG2, HFS, and Vero with IC_50_ values of 26.03, 15.92, 10.08, 5.29, 10.97, and 17.12 µg/mL, respectively [12].

### 2.4. Biocidal Influence of Uv@Ag-NPs

**Figure 12:**
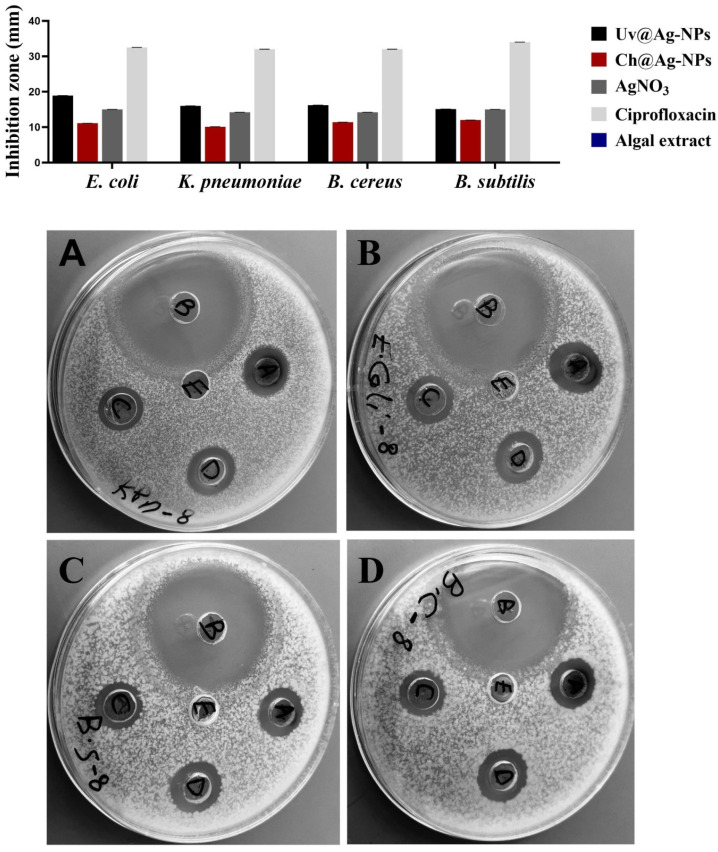
Inhibitory activities of 1 mg/mL of Uv@Ag-NPs, Ch@Ag-NPs, AgNO_3_, algal extract, and ciprofloxacin against *E. coli*, *K. pneumoniae*, *B. cereus*, and *B. subtilis*. Letters written on well refer to (A) Uv@Ag-NPs, (B) ciprofloxacin, (C) Ch@Ag-NPs, (D) AgNO_3_, and (E) algal extract.

The inhibitory effects of Uv@Ag-NPs, Ch@Ag-NPs, AgNO_3_, the algal extract, and ciprofloxacin against *E. coli*, *K. pneumoniae*, *B. cereus*, and *B. subtilis* were examined using the agar well diffusion method. After excluding ciprofloxacin, the data revealed that Uv@Ag-NPs had the highest biocidal activity against the tested microbes (Figure 12 and Table 3). The Uv@Ag-NPs showed greater activity against Gram-negative than Gram-positive bacteria. *E. coli* was the most sensitive microorganism to Uv@Ag-NPs with an IZ of 18.9 ± 0.03 mm, while *B. subtilis* showed the lowest response against Uv@Ag-NPs with an IZ of 15.1 ± 0.04 mm. The positive charge of Ag-NPs plays a significant role in enhancing their antibacterial activity via electro-attractive interactions between the negatively charged NPs and bacterial membranes [60]. Here, the Uv@Ag-NPs showed unexpected results; they had a negative charge on their surface but showed marked activity against Gram-negative bacteria, suggesting that the role of the charge in enhancing the biocidal efficiency of Uv@Ag-NPs can be overlooked. However, the biocidal activity of Uv@Ag-NPs against the tested bacteria can be attributed to their small size and large surface area and the surface chemistry of these NPs facilitating their interactions with cellular membranes and components inhibiting bacterial growth. The biocidal activity of Ag-NPs was highly dependent on the nanosize; the smaller sizes with larger surface areas allowed better contact with the cell membrane [61,62]. The Ch@Ag-NPs (negatively charged NPs), relative to Uv@Ag-NPs and AgNO_3,_ had the lowest inhibition zone; higher values (12.0 ± 0.01 mm) were recorded for *B. subtilis*, while the lower IZ was 10.1 ± 0.03 mm for *K. pneumoniae.* The lower biocidal activity of Ch@Ag-NPs compared to Uv@Ag-NPs could be attributed to the large nanosize of Ch@Ag-NPs trapping the NPs outside the bacterial wall, tackling their entrance into cells and reducing their activity against the bacterial cells. Moreover, the absence of functional groups around Ch@Ag-NPs’ surface may also substantially impact the therapeutic action of Ch@Ag-NPs. It was found that the functional groups on the NPs surface mitigate their biological activity and toxicity via their interaction with cellular structures and biomolecular corona [28].

The highest IZ values of AgNO_3_ were for both *E. coli* and *B. subtilis* at 15.0 ± 0.13 and 15.0 ± 0.19 mm, respectively, while the lower values were for both *K. pneumoniae* and *B. cereus* with values of 14.2 ± 0.45 and 14.2 ± 0.03 mm, respectively. Intriguingly, 1 mg/mL of Uv@Ag-NPs and AgNO_3_ resulted in a similar IZD value (about 15.0 mm) against *B. subtilis*. These data suggested that the biocidal activity of Uv@Ag-NPs against *B. subtilis* might be due to the nature of Ag ions rather than the algal functional groups, which might have other roles such as stabilising and charging NPs or directing the NPs to bacterial cells. Moreover, 1 mg/mL of algal extract was not enough to inhibit the bacterial growth with zero IZ against all tested microbes. These data show that Uv@Ag-NPs exhibited marked biocidal activity against the tested microbes compared with silver nitrate and Ch@Ag-NPs, suggesting that the small size with high specific surface area and functional group coating of the Uv@Ag-NPs have a significant influence on their biological activities. Ag-NPs (with a particle size of 4.06 nm) synthesised using *pu-erh tea* leaf extract inhibited the growth of *E. coli*, *K. pneumoniae*, *Salmonella Typhimurium*, and *Salmonella Enteritidis* with IZ values of 15, 10, 20, and 20 mm, respectively [63], while the Ag-NPs (with spherical shape and diameter range of 4.5 to 26 nm) synthesised from *Desertifilum IPPAS B-1220* showed antibacterial activity against *B. cereus* and *B. subtilis* with IZs of 16.33 ± 0.33 and 17.33 ± 0.33 mm, respectively [64].

## 3. Materials and Methods

### 3.1. Materials

Silver nitrate (AgNO_3_); chemically synthesised NPs with nanosizes of <100 nm, spherical shapes, and 99.5% purity; and 3-(4,5-dimethylthiazol-2-yl)-2,5-diphenyltetrazolium bromide) tetrazolium reduction assay (MTT) assay and 5-fluorouracil (5-FU) were purchased from Sigma-Aldrich (St. Louis, MO, USA). The cell culture tools and media were purchased from Gibco (Thermo Fisher Scientific, Waltham, MA, USA). PC3, MDA-MB-231, T47D, MCF-7, and HFs cells were purchased from Nawah Scientific company, Egypt, who obtained the cells from the American Type Culture Collection (ATCC, Manassas, VA, USA), and microbial isolates were obtained from the Department of Microbiology, King Saud University, Riyadh, Saudi Arabia.

### 3.2. Unicellular ulvophyte sp. MBIC105

#### 3.2.1. Isolation and Morphological Estimation

The microalgae were isolated from muddy soil in Riyadh, Saudi Arabia, using the serial dilution method reported by Hamida et al. [12]. The microalgae were kept in a sterile BG-11 media-containing flask in an incubator under a fluorescence lamp (2000 ± 200 Lux) with a 12:12 h dark/light cycle at room temperature for 15 days. Inverted, light, and scanning electron microscopes were used to determine the morphological appearance and purity of the microalgae. The sample was washed at least three times with water and ethanol and fixed in 70% ethanol. A small volume of the algal suspension was loaded onto a sterile glass piece fixed on a carbon paste attached to a copper stub. The sample was subsequently coated with a platinum coater (JEC-3000FC, Joel, Tokyo, Japan) for 80 s for scanning electron microscopy (SEM) (JSM-IT500HR, Joel, Japan) at 15 kV.

#### 3.2.2. 18s rRNA Identification

The sample was identified using 18s rRNA identification. The DNA was extracted as described by Hamida et al. [12]. The PCR step with specific primers (forward primer: CCAGCAGCCGCGGTAATTCC; reverse primer: ACTTTCGTTCTTGATTAA) was performed to amplify the extracted DNA for sequencing using an ABI 3730 DNA sequencer (Thermo Fisher Scientific, USA).

#### 3.2.3. Gas Chromatography–Mass Spectrometry (GC-MS) Analysis

The volatile components in the algal aqueous extract were screened using the Trace GC-TSQ mass spectrometer (Thermo Fisher Scientific, Austin, TX, USA) with the direct capillary column TG–5MS (30 m × 0.25 mm × 0.25 μm film thickness). Briefly, 50 mg of algal powder was soaked in 50 mL of boiled distilled water (dist. H_2_O) and sonicated for 30 min. Subsequently, the sample was allowed to macerate for 24 h, followed by filtration with a syringe filter (0.22 μm). The filtrate was dried in a vacuum oven at 50 °C for 48 h. The temperature of the column oven was 50 °C initially before it was increased at a rate of 5 °C/min to 250 °C, maintained for 2 min, increased to 300 °C at a rate of 30 °C/min, and maintained for 2 more min. The injector and MS temperatures were maintained at 270 and 260 °C, respectively. Helium was utilised as a carrier gas at a constant flow rate of 1 mL/min. The solvent delay was 4 min, and diluted samples of 1 μL were injected automatically using an Autosampler AS1300 coupled with GC in the split mode. Electron ionisation mass spectra were collected at 70 eV ionisation voltages over the range of 50–650 *m*/*z* in full scan mode. The ion source temperature was set to 200 °C. Components of the algal extract were identified by comparing their mass spectra with those of the WILEY 09 and NIST 14 mass spectral databases [12].

#### 3.2.4. Algal Aqueous Extract Preparation

The microalgae biomass was collected by centrifugation at 4700 rpm for 10 min, washed more than thrice with dist. H_2_O, and lyophilised for 24 h using LYOTRAP (LTE Scientific, Greenfield, U.K.). The algal watery extract was prepared by dissolving an equal amount of algal powder with dist. H_2_O and boiling at 80 °C for 30 min. Subsequently, the algal extract was spun at 4700 rpm for 10 min, and the supernatant was filtered using Whatman filter paper No. 1. The filtrate was used freshly to synthesise the Ag-NPs (Uv@Ag-NPs) [12].

### 3.3. Uv@Ag-NPs Synthesis

#### 3.3.1. Optimisation Parameters for the Biofabrication of Uv@Ag-NPs

To determine the optimum conditions for Uv@Ag-NP biofabrication, various parameters were screened.

##### Precursor Concentrations and Ratios

Uv@Ag-NPs were produced with various concentrations (1, 2, 5, and 10 mM) of silver nitrate at a constant ratio of 1 to 9 (algal extract to silver nitrate) and a temperature of 25 °C under illumination for 24 h. Two millilitres of the synthesised Uv@Ag-NPs at each concentration was screened using UV spectroscopy (Shimadzu, Japan). After obtaining the optimum concentration, the effects of various ratios of precursor and algal extracts were determined. Four ratios were tested by mixing algal extract with 1 mM of AgNO_3_ at ratios of 1:1, 1:2, 1:4, and 1:9, respectively, under the same constant conditions.

##### Temperature and pH

To estimate the influence of temperature and pH on NP biofabrication, 100 mL of 1 mM of AgNO_3_ was mixed with 100 mL of algal extract and exposed to various temperatures of 25, 40, 60, and 100 °C for 1 h under other constant conditions. At the optimum temperature (100 °C), the pH values of AgNO_3_ and the algal extract mixture were adjusted dropwise using 0.1 M hydrochloric acid or sodium hydroxide to 5, 7, 8, 9, and 12 under the same constant conditions for synthesis.

##### Illumination and Incubation Duration

Briefly, 100 mL of 1 mM of AgNO_3_ was mixed with 100 mL of algal extract at 100 °C and a pH of 7. The mixture was incubated once in the dark and once in light (fluorescence lamp with 2000 ± 200 Lux) for 24 h. An aliquot was measured using UV spectroscopy to determine the optimum illumination conditions. The influence of the incubation duration was subsequently estimated by incubating the AgNO_3_ and algal extract mixture under light conditions for 24, 48, and 72 h.

After obtaining the optimum conditions for biosynthesising the Uv@Ag-NPs, the NPs were synthesised on a large scale (5 L), centrifuged at 12,000 rpm for 15 min, washed at least thrice with dist. H_2_O, and lyophilised for 8 h. The powder NPs were weighed and collected in sterile Eppendorf for further experiments.

### 3.4. Characterisation of Uv@Ag-NPs

#### 3.4.1. UV Spectroscopy

For each optimum parameter, an aliquot (2 mL) of Uv@Ag-NPs was screened using UV spectroscopy for a wavelength range of 200–800 nm and a resolution of 1 nm.

#### 3.4.2. Morphological and Elemental Composition Analysis of Uv@Ag-NPs

The shapes, sizes, elemental compositions, and distributions of the Uv@Ag-NPs were analysed using a high-resolution transmission electron microscope (HR-TEM), TEM, and SEM combined by an energy dispersive X-Ray analysis (EDx) detector. The Uv@Ag-NPs were collected by centrifugation at 12,000 rpm for 15 min, washed at least thrice with dist. H_2_O and ethanol, and suspended in 1 mL ethanol and sonicated for 15 min. For imaging, 20 µL of the NP suspension was dropped onto the carbon-coated copper grid and air-dried to be examined by TEM (JEM-1400Flash, Joel, Tokyo, Japan) at 120 kV. Similarly, for SEM, 20 µL of the NP suspension was loaded on a sterile glass attached to a copper stub and air-dried. The sample was coated with platinum and examined at 15 kV using SEM. On the other hand, a small amount of powdered Uv@Ag-NPs was loaded onto carbon paste attached to a copper stub and coated with platinum for 80 sec to be analysed with an EDx detector (JSMIT500HR, STD-PC80, Joel, Tokyo, Japan) [12].

#### 3.4.3. Fourier Transform Infrared Spectroscopy (FTIR) and Zeta Sizer

The surface chemistry of the Uv@Ag-NPs powders was detected in a range of 400–4000 cm^−1^ using FTIR spectroscopy (Shimadzu, Kyoto, Japan). The potential charges and hydrodynamic diameter of the Uv@Ag-NPs were determined by sonicating the NP suspension (500 µg/mL) for 15 min, diluting it 10-fold, sonicating for 1 to 2 min, and transferring it to Utype tubes at 25 °C for measurement using the zeta sizer (Malvern, U.K.).

### 3.5. Anticancer Activity

The antiproliferative activities of Uv@Ag-NPs, Ch@Ag-NPs, and 5-FU (as positive controls) were screened against four malignant cell lines, namely PC3, MCF-7, MDA-MB-231, and T47D, and one normal cell line, HFs, using the MTT kit. In brief, a cell density of 5 × 10^4^ cells/mL was seeded onto a 96-well plate and incubated in a 5% CO_2_ incubator for 24 h at 37 °C. At 75% confluency, the cells were subjected to serial dilution of Uv@Ag-NPs (200, 100, 50, 25, 12.5, 3.1, and 1.6 µg/mL), while the concentrations of both Ch@Ag-NPs and 5-FU were 1000, 500, 250, 125, 62.5, 31.25, 15.62, 7.81, and 3.90 μg/mL. Uv@Ag-NPs, Ch@Ag-NPs, and 5-FU were suspended in DMEM media, and the NP suspension was sonicated for 15 min. The 5-FU mixture was vortexed for 1 min. All mixtures were filtered using a 0.45 μm syringe filter for direct application to cells. The treated plates were incubated for 24 h in a 5% CO_2_ incubator at 37 °C. After incubation, the media were discarded and replaced with 100 μL/well fresh media, and 10 μL/well of MTT solution (5 mg of MTT powder dissolved in 1 mL of sterile PBS, vortexed until dissolution, and filtered using a syringe filter) was added. The plates were incubated for 4 h, and the media was removed. Subsequently, 100 μL/well of DMSO was applied, and the plates were shacked at 400 rpm for 15 min to dissolve the formazan dye crystal. The plates were read on a Hercules, CA, USA) at 570 nm [3]. Cell viability (%) was estimated according to the following equation:

(Abs(treated)/(Abs(control)) × 100

The IC_50_ (half-maximal growth inhibitory concentration) was calculated using a sigmoidal curve.

### 3.6. Antibacterial Activity

*Escherichia coli ATCC8739*, *Klebsiella pneumoniae ATCC13883*, *Bacillus cereus ATCC9634*, and *Bacillus subtilis ATCC6633* were cultured in nutrient broth for up to 18 h at 37 °C and maintained through continuous subculturing in broth and on solid media. The inhibitory activities of 1 mg/mL of Uv@Ag-NPs, Ch@Ag-NPs, AgNO_3_, algal extract, and 5 µg/mL ciprofloxacin were assessed against the tested bacteria using the agar well diffusion method. In brief, 4 mL of the bacterial strain was suspended in 50 mL of nutrient agar media. The mixture was poured into sterilised Petri dishes and dried at 37 °C. Four 8 mm wells were created in the agar plates using a cork borer. Subsequently, 100 μL of Uv@Ag-NPs, Ch@Ag-NPs, AgNO_3_, algal extract, and ciprofloxacin suspensions were poured into the 8 mm wells. The plates were kept in a bacterial incubator at 37 °C for 24 h. Ch@Ag-NPs and ciprofloxacin were used as positive controls, while dist. H_2_O was used as a negative control. The inhibition zone (IZ) was estimated after 24 h using a transparent ruler [65].

### 3.7. Statistical Analysis

All experiments were performed in triplicate independently, and the data are presented as mean ± SEM. One-way analysis of variance (ANOVA) was performed to compare differences between untreated and treated groups using graphPrism version 9.3.1 (GraphPad Software Inc., San Diego, CA, USA); *p* < 0.05 was considered statistically significant. For characterisation analysis of Uv@Ag-NPs, origin 8 (OriginLab Corporation, Northampton, MA, USA) and ImageJ (National Institutes of Health, Bethesda, MD, USA) were utilised.

## 4. Conclusions

These findings provide, for the first time, information about the novel microalgae unicellular *ulvophyte* sp. MBIC10591 and their potential for Ag-NP biogenesis. Herein, we report the morphological appearance of the unicellular *ulvophyte* sp. MBIC10591; the cells appeared spherical with cup-shaped spongiomorph chloroplasts with pyrenoids surrounded by several starch grains. Single cells were dominantly distributed; however, package cells with parenchyma-like structures containing more daughter cells were also found. The unicellular *ulvophyte* sp. MBIC10591 is enriched with various biomolecules, including vitamins, antioxidants, amino-acid-like compounds, organosulphur compounds, fatty acids, hydrocarbons, polysaccharides, phenol, and alcohols and may be a source of several therapeutic compounds. More investigations are needed to identify several molecules in different organic extracts of the unicellular *ulvophyte* sp. MBIC10591. These biomolecules enable the unicellular *ulvophyte* sp. MBIC10591 to biosynthesise small Ag-NPs. The Uv@Ag-NPs have UV–Vis spectra at 409.5 nm with spherical and cubic shapes. It was found that the optimum conditions for synthesising Uv@Ag-NPs include 1 mM AgNO_3_, a ratio of 1:1 for AgNO_3_ and algal extract, temperature of 100 °C for 1 h, and pH of 7 under light conditions for 24 h. The nanosize of these NPs was 5–60 nm with an average diameter of 12.1 ± 1.2 nm, while their HD and ZP were 178.1 nm with polydispersity index of 0.38 and −26.7 mV, respectively; these suggest their polydispersity and colloidal stability. Several functional groups were detected on Ag-NP surfaces. Proteins or/and polysaccharides or/and alcohols are responsible for reducing Ag-NPs, while fatty acids or/and hydrocarbons are the stabilising agents responsible for preventing the agglomeration of Ag-NPs. Uv@Ag-NPs exhibited marked anticancer activity against prostate cancer and multidrug resistance breast cancers with low toxicity against HFs. They also demonstrated marked inhibitory activity against Gram-negative bacteria; *E. coli* was the most susceptible to NPs, while *B. subtilis* was the most resistant. These antiproliferative activities and biocidal effects of Uv@Ag-NPs may be attributed to their unique physicochemical characteristics including their small sizes, large areas, shapes, and surface chemistry, which allow them to adsorb on cell surfaces, penetrate membranes and increase the permeability of outside walls or biomolecules such as proteins and enzymes, and interact with cellular organelles and biomolecules causing cellular dysfunction and cell death. Further study of the chemistry of the unicellular *ulvophyte* sp. MBIC10591 is recommended to discover more metabolites that can serve as drugs. Moreover, more optimisation parameters are needed to obtain more uniform shapes of Uv@Ag-NPs and assays to explore their biological activities and mechanisms inside malignant and microbial cells.

## Figures and Tables

**Table 1:** Chemical composition analysis of the unicellular *ulvophyte* sp. MBIC10591 using GC-MS.

No.	Biomolecule Name	Retention Time	Area %	Mentioned Factor	Molecular Formula	Molecular Weight
1	D-fructose, diethyl mercaptal, pentaacetate	4.04, 4.09, 16.34	0.49, 0.63, 3.25	659, 655, 659	C_20_H_32_O_10_S_2_	496
2	Methyl 4,7,10,13-hexadecatetraenoate	6.15	0.89	708	C_17_H_26_O_2_	262
3	2-Aminoethanethiolsulphuric acid	6.99, 11.39, 21.7	1.10, 0.96, 0.71	699, 727, 760	C_2_H_7_NO_3_S_2_	157
4	25,26,27-Trinorcholecalcifer-24-al	7.34	2.05	766	C_24_H_36_O_2_	356
5	Trisulfide, di-2-propenyl	12.19	3.34	760	C_6_H_10_S_3_	178
6	1,2-Diacetin	13.28	9.63	927	C_7_H_12_O_5_	176
7	[5,5-dimethyl-6-(3-methyl-buta-1,3-dienyl)-7-oxa-bicyclo [4.1.0]hept-1-yl]-methanol	16.59	1.22	748	C_14_H_22_O_2_	222
8	3a,4,7,7a-Tetrahydrodimethyl-4,7-methanoindene	17.26	1.58	775	C_12_H_16_	160
9	Phenol, 2,6-bis(1,1-dimethylethyl)-	17.45	2.06	749	C_14_H_22_O	206
10	Methyl 6,9-octadecadiynoate	18.83	0.94	739	C_19_H_30_O_2_	290
11	1H-Cycloprop[e]azulen-7-ol, decahydro-1,1,7-trimethyl-4-methylene-, [1ar-(1aalpha,4aalpha,7beta,7abeta,7balpha)]-	18.93	3.61	899	C_15_H_24_O	220
12	3-Oxo-20-methyl-11-à-hydroxyconanine-1,4-diene	19.47	0.73	757	C_22_H_31_NO_2_	341
13	2,5-Octadecadiynoic acid, methyl Ester	20.72	0.76	786	C_19_H_30_O_2_	290
14	(5e,7e)-9,10-Secocholesta-5,7,10-triene-3,25,26-triol #	22.23	0.64	753	C_27_H_44_O_3_	416
15	9-Oximino-2,7-diethoxyfluorene	25.92, 27.92	1.43, 1.75	745, 747	C_17_H_17_NO_3_	283
16	Methyl 14-methylpentadecanoate	26.39	4.38	742	C_17_H_34_O_2_	270
17	1-Heptatriacotanol	29.52	0.85	780	C_37_H_76_O	536
18	9-Octadecenoic acid (z)-, 2-Hydroxy-1-(hydroxymethyl)ethyl Ester	29.67	3.40	792	C_21_H_40_O_4_	356
19	Cyclopropanebutanoic acid, 2-[[2-[[2-[(2 pentylcyclopropyl)meth yl]cyclopropyl]methyl]cyclopropyl] methyl]-, methyl ester	30.18	1.23	813	C_25_H_42_O_2_	374
20	9-(2′,2′-Dimethylpropanoilhydrazono)-3,6-dichloro-2,7-bis-[2-(diethylamino)-ethoxy]fluorene	30.89	5.84	761	C_30_H_42_Cl_2_N_4_O_3_	576
21	1,2-Benzenedicarboxylic acid	32.70, 34.07, 35.15, 36.85, 36.07	7.78, 3.83, 4.94, 6.02, 12.89	789, 783, 776, 795, 779	C_24_H_38_O_4_	390
22	Widdrol hydroxyether	33.12, 33.19	3.17, 5.39	770, 752	C_15_H_26_O_2_	238
23	9,12,15-octadecatrienoic acid, 2,3-bis [(trimethylsilyl)oxy]propyl ester, (z,z,z)	35.31	1.25	734	C_27_H_52_O_4_Si_2_	496
24	Tetraneurin-a-diol	35.73	0.71	805	C_15_H_20_O_5_	280

**Table 2:** EDx analysis of Uv@Ag-NPs synthesised from the unicellular *ulvophyte* sp. MBIC10591.

Element	Line	Mass%	Atom%
C	K	6.93 ± 0.02	32.29 ± 0.09
O	K	1.81 ± 0.02	6.32 ± 0.08
Al	K	0.3 ± 0.01	0.63 ± 0.02
Cl	K	12.18 ± 0.03	19.22 ± 0.05
Cu	K	1.13 ± 0.04	1.00 ± 0.04
Zn	K	0.91 ± 0.05	0.78 ± 0.04
Ag	L	76.74 ± 0.11	39.78 ± 0.06
Total		100	100

**Table 3:** Inhibition zones of 1 mg/mL of Uv@Ag-NPs, Ch@Ag-NPs, AgNO_3_, algal extract, and ciprofloxacin for *E. coli*, *K. pneumoniae*, *B. cereus*, and *B. subtilis*.

Microorganisms	Drugs (µg/mL)				
	Uv@Ag-NPs	Ch@Ag-NPs	AgNO_3_	Algal Extract	Ciprofloxacin
	IZD (mm)				
*E. coli*	18.9 ± 0.03	11.1 ± 0.14	15.0 ± 0.13	0.0 ± 0.0	32.5 ± 0.03
*K. pneumoniae*	16.0 ±0.01	10.1 ± 0.03	14.2 ± 0.45	0.0 ± 0.0	32.0 ± 0.03
*B. cereus*	16.2 ± 0.18	11.4 ± 0.05	14.2 ± 0.03	0.0 ± 0.0	32.0 ± 0.06
*B. subtilis*	15.1 ± 0.04	12.0 ± 0.01	15.0 ± 0.19	0.0 ± 0.0	34.0 ± 0.03

## Data Availability

Additional data to those presented here are available from the corresponding author upon reasonable request.

## Sample Availability

Samples of the algae or nanoparticles are available from the authors.
